# Case Report: Solitary adrenal metastasis from esophageal adenocarcinoma

**DOI:** 10.3389/fmed.2025.1623443

**Published:** 2025-07-17

**Authors:** Riddhi Machchhar, Ahmed Dawood Al Mahrizi, Fatima Mossolem, Mariam Abdeen, Ujjwala Jain, Jyotibala Jain, Prashant Desai

**Affiliations:** ^1^Department of Graduate Medical Education, Ocean University Medical Center, Brick Township, NJ, United States; ^2^Faculty of Medicine & Surgery, University of Malta, Msida, Malta; ^3^Rowan University School of Osteopathic Medicine, Stratford, NJ, United States; ^4^Department of Graduate Medical Education, Tufts University School of Medicine, Boston, MA, United States

**Keywords:** esophageal adenocarcinoma, adrenal metastasis, systemic therapy, FOLFOX, palliative care

## Abstract

**Introduction:**

Solitary adrenal metastasis from a primary esophageal malignancy is relatively rare. While there are case reports of aggressive treatment with esophagectomy and adrenalectomy providing long-term survival, the treatment paradigm is not well defined. Complications from esophageal adenocarcinoma and its treatment can significantly impact the patient’s quality of life and prognosis.

**Case presentation:**

Our patient was treated with systemic therapy and, although he initially had a complete response, he later experienced local disease progression along with the development of additional metastatic sites. The patient began FOLFOX, a standard chemotherapy regimen for esophageal/gastric cancer. He experienced side effects such as fever, malaise, and constipation, which were symptomatically managed. Given these side effects, the patient’s FOLFOX regimen underwent a 25% dose reduction of the 5FU bolus. A PET/CT scan after three months showed a marked response to chemotherapy, with complete resolution of detectable disease. The patient reported fatigue, bone pain managed with Neulasta, nausea controlled with antiemetics, and neuropathy in his feet. The patient then began a new chemotherapy regimen with Taxol/Ramucirumab with dose modifications in response to side effects. Continued adjustments to the treatment and dosages were made for progressive side effects and the patient elected to receive palliative radiation to the esophagus, along with holistic supportive care. The treatment plan shifted to palliative care, focusing on quality of life, rather than curative, due to the complexities of his cancer.

**Conclusion:**

While aggressive treatments may offer hope for a cure in select patients with isolated adrenal metastasis from esophageal cancer, the general approach should remain cautious, with systemic therapy as the first line of defense. This case highlights the need for careful selection to identify patients who may benefit from aggressive surgical treatment. Ongoing research and clinical trials are needed to better define treatment protocols and improve outcomes for this challenging patient group.

## Introduction

Esophageal adenocarcinoma is a malignancy originating from the glandular tissue of the esophagus, and is characterized by its aggressive progression and poor prognosis; only 16% of patients survive 5 years after diagnosis and the median survival time is less than 1 year (1). The disease course often begins with Barrett’s esophagus, which is associated with lifestyle factors such as diet, obesity, and gastroesophageal reflux disease (2). Pathology evolves from normal histology to dysplasia and metaplasia, and eventually adenocarcinoma, with the potential to metastasize to distant organs, including the adrenal glands (3).

The treatment of esophageal adenocarcinoma is multifaceted and depends on the stage of the disease (4). Early-stage cancers may be treated with surgery alone, while advanced stages often require a combination of chemotherapy, radiation therapy, and surgery (4). Immunotherapy has also emerged as a treatment option, particularly for cases that do not respond to conventional therapies (5). The approach to treating esophageal adenocarcinoma has traditionally centered around surgery, especially in cases where the cancer is localized and resectable (6). However, for cases with distant metastasis, such as to the adrenal glands, chemotherapy, often in combination with radiation therapy, is typically employed (6). Adrenalectomy may be considered when metastasis is confined to the adrenal gland and the primary cancer is either well-controlled or resectable (6).

Complications from esophageal adenocarcinoma and its treatment can significantly impact the patient’s quality of life and prognosis (7). These may include difficulty swallowing, bleeding, pain, and blockages in the esophagus (7). This case report of solitary adrenal metastasis from esophageal adenocarcinoma underscores the rarity of such metastatic presentations and highlights the importance of a comprehensive approach to diagnosis and management (7).

## Case description

A 66-year-old male veteran of the Vietnam war with a past medical history of coronary artery disease and hyperlipidemia presented with progressive dysphagia, initially to solid food and later to liquids, accompanied by odynophagia. He reported a long-standing sensation of a lump in his chest, food regurgitation, and heartburn. The patient had no history of cigarette smoking or alcohol use and no significant family history.

**Figure 1:**
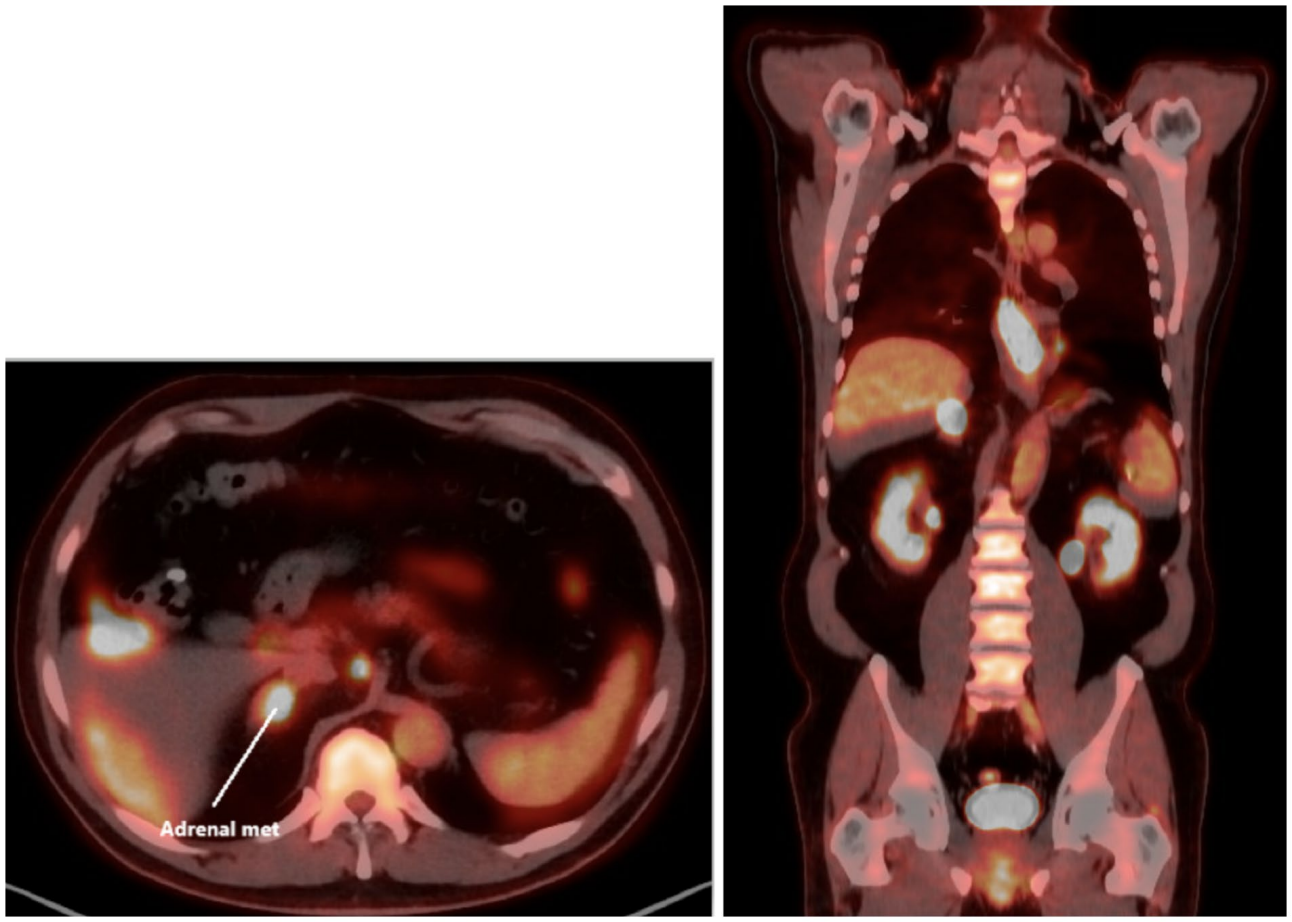
Adrenal metastasis on axial PET scan before treatment (left) and initial PET coronal image before systemic therapy shows esophageal and adrenal lesions (right).

**Figure 2:**
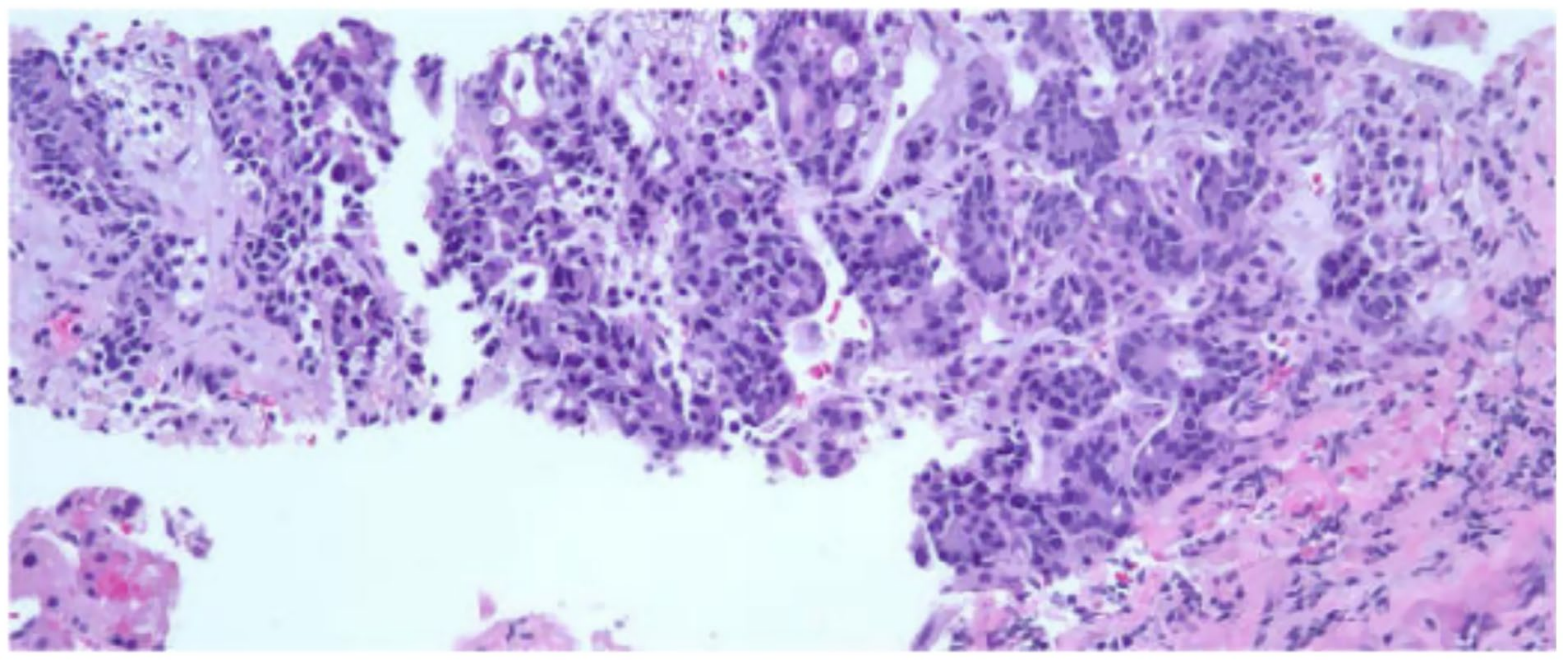
The adrenal gland tissue exhibits malignant glands within a desmoplastic stroma. The malignant cells exhibit pleomorphic nuclei with vesicular chromatin, prominent nucleoli and moderate amounts of amphophilic cytoplasm. Immunoperoxidase studies performed demonstrate the malignant cells to be positive for AE1/AE3, CK7, CDX2 and weakly positive for CK20. The Tumor Cells are negative for PAX8, NKX3.1, TTF-1 and GATA3. This immunohistochemical staining profile is consistent with foregut midgut primary and the pancreaticobiliary tree. Patient has a history of esophageal adenocarcinoma and this metastasis is consistent with metastatic esophageal adenocarcinoma.

Initial assessments included a review of the patient’s medical history, blood work, and imaging (Supplementary materials). Diagnostic procedures revealed gastritis and an esophageal mass in the lower third of the esophagus, with a biopsy confirming moderately to poorly differentiated esophageal gastroesophageal junction adenocarcinoma. A colonoscopy identified tubular adenomas, and a CT scan showed abnormal lymph nodes near the distal esophagus, a mass in the right adrenal gland suggestive of possible metastasis, an enlarged prostate, and parapelvic renal cysts prompting further evaluation with a PET scan (Figure 1) and an IR-guided biopsy. Biopsy of the right adrenal gland confirmed metastatic adenocarcinoma, most consistent with a foregut/midgut primary origin (Figure 2).

**Figure 3:**
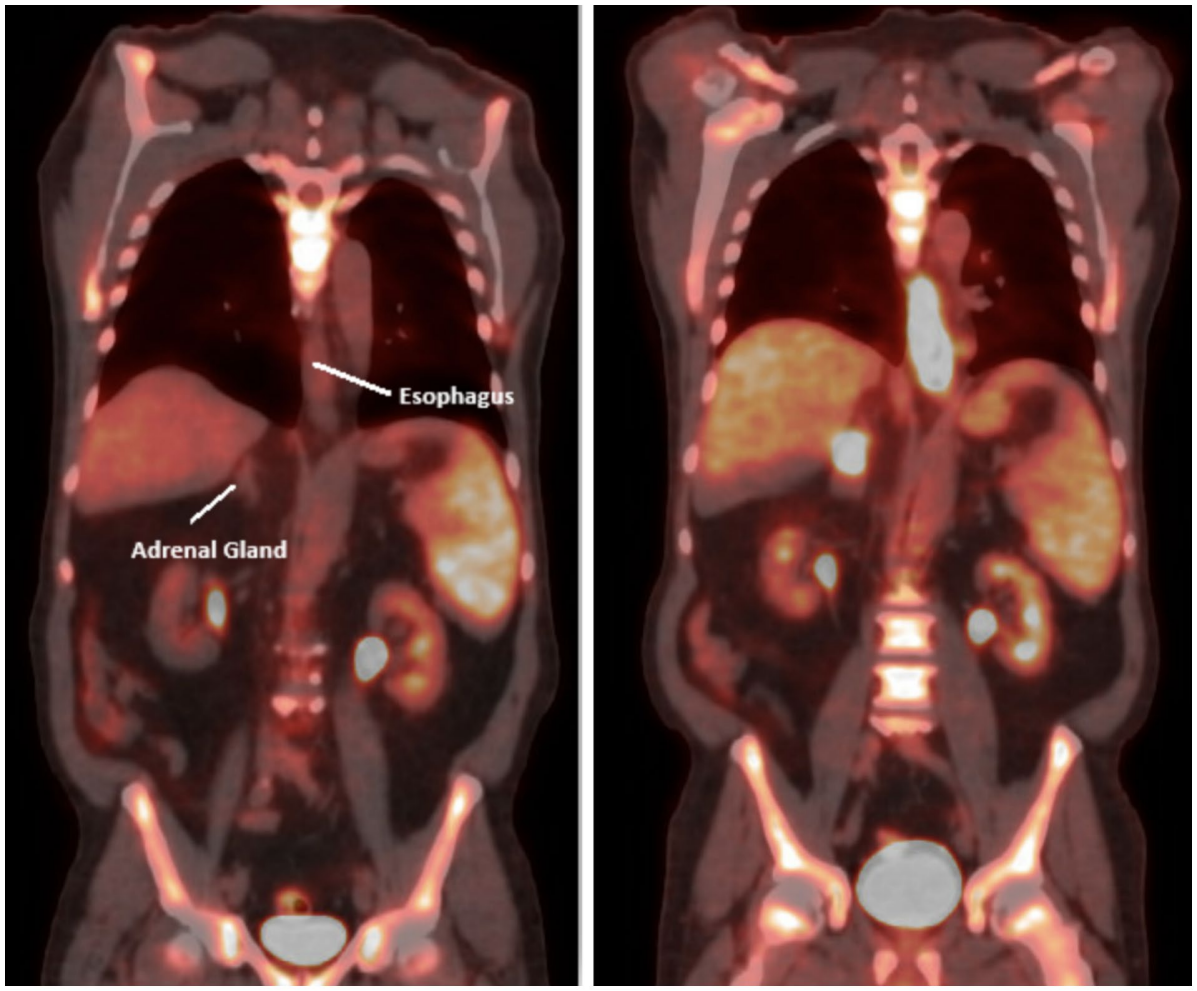
PET scan coronal view after 4–5 cycles of chemotherapy (left) showing interval decrease in the circumferential thickening and FDG activity associated with the distal 3rd of the thoracic esophagus and repeat PET scan after 2 additional cycles showed significant progression of disease both in the esophagus and adrenals (right).

The patient began FOLFOX, a standard chemotherapy regimen for esophageal/gastric cancer. The regimen included fluorouracil, leucovorin, and OXALIplatin, supplemented with dexamethasone. He experienced side effects such as fever, malaise, and constipation, which were symptomatically managed. Given these side effects, the patient’s FOLFOX regimen underwent a 25% dose reduction of the 5FU bolus. Follow-up visits before cycles 6 and 10 confirmed the patient’s ability to continue treatment, albeit with further dose reductions and modifications due to thrombocytopenia and neuropathy. A PET/CT scan after three months showed a marked response to chemotherapy, with complete resolution of detectable disease (Figure 3). The patient reported fatigue, bone pain managed with Neulasta, nausea controlled with antiemetics, and neuropathy in his feet (see Table 1).

**Table 1:** Timeline table.

Time point	Clinical findings & interventions
Initial presentation (week 0)	Progressive dysphagia to solids → liquids, odynophagia, chest lump sensation, heartburn → blood work, upper endoscopy with biopsy
Diagnostic workup (week 1)	CT chest/abdomen: distal esophageal mass, right adrenal nodule → PET/CT scan → IR-guided adrenal biopsy confirms metastatic adenocarcinoma
Treatment initiation (week 2)	FOLFOX chemotherapy (5FU, leucovorin, oxaliplatin) + dexamethasone; initial 25% dose reduction for 5FU bolus
Interim response (week 14)	PET/CT after 3 months: complete metabolic response
Dose adjustments (weeks 14–24)	Further dose modifications for thrombocytopenia and neuropathy; continued cycles through cycle 8
Disease progression (week 25)	PET/CT: new supraclavicular, mediastinal, para-esophageal nodes; recurrent adrenal uptake
Second-line therapy (week 26)	Taxol + ramucirumab with esophageal radiation for dysphagia
Further management (week 30)	Keytruda immunotherapy; stereotactic radiotherapy to adrenal gland
Shift to palliation (week 32)	Grade 2 neutropenia, neuropathy, elevated bilirubin → focus on palliative radiation and supportive care

It was decided to give an additional 2 cycles of chemotherapy due to its encouraging effect and to target undetectable cells. However, a repeat PET scan after 2 additional cycles indicated disease progression (Figure 2). The scan highlighted several areas of concern, including a right supraclavicular lymph node, focal thickening of the distal esophageal wall, and nodes in the mediastinum and paraesophageal regions, all with increased uptake indicating hypermetabolic activity. The right adrenal nodule and a retroperitoneal lymph node also showed increased uptake. The patient then began a new chemotherapy regimen with Taxol/Ramucirumab with dose modifications in response to side effects. Radiation therapy to the esophagus was also initiated to address dysphagia.

As the treatment progressed, the patient experienced grade 2 neutropenia and elevated bilirubin levels, necessitating further chemotherapy dose adjustments Oxaliplatin was switched to Abraxane due to progressive neuropathy. The treatment plan included a cycle of Keytruda, following mixed responses on PET/CT and subsequent stereotactic radiosurgery (SRS) to the right adrenal gland. Continued adjustments to the treatment and dosages were made for progressive side effects and the patient elected to receive palliative radiation to the esophagus, along with holistic supportive care. The treatment plan shifted to palliative care, focusing on quality of life, rather than curative, due to the complexities of his cancer.

## Discussion

Patients with isolated adrenal metastasis from an esophageal primary cancer present a unique challenge in oncological management (8). The rarity of such cases means that there is no consensus on the optimal treatment strategy (8). Some clinicians advocate for an aggressive approach with curative intent, which may include esophagectomy and adrenalectomy (9). This is supported by a handful of case reports demonstrating long-term survival following such interventions (10–12). However, these cases are exceptional and not indicative of the general prognosis for this patient population.

The lack of a well-defined treatment paradigm leads most oncologists to favor initial systemic therapy (13). The rationale behind this approach is to control systemic disease and assess the tumor’s responsiveness to chemotherapy before considering more invasive procedures (13). FOLFOX is commonly used in this context due to its efficacy in gastrointestinal cancers (11). After 6 cycles of FOLFOX, our patient showed a complete response on PET scan, which was encouraging. The plan was to complete 8 cycles before transitioning to local therapy. However, a PET scan after 8 cycles revealed disease progression, including recurrence of adrenal metastasis and new metastatic sites. In similar cases where surgery is performed, median survival has been reported at 36 months from diagnosis (11). Adrenal metastasis were commonly identified on the following imaging modalities: CT, PET, MRI or after a postoperative follow-up from an endoscopy for the primary site (11, 12).

This progression despite systemic therapy highlights the aggressive nature of esophageal cancer and its propensity for metastasis (11). Moreover, patients with recurrent esophageal carcinoma have an average survival of 4–6 months (11). Furthermore, as seen in our patient’s complicated management, malignant adrenal gland tumors secondary to esophageal carcinoma are rare with eight cases reported between 1995 and 2014 (11) while another study reported a 3% occurrence and a 12% on autopsy (12). This metastasis was observed to favor the male gender with a median age of 63, and currently lacks a defined algorithm of care (11). It underscores the need for continuous evaluation and adjustment of treatment plans based on disease dynamics (14, 15). The case also emphasizes the importance of considering palliative care options early in the treatment process, as the goal shifts from curative to quality-of-life improvement.

Dellaportas et al. (16) and Hadlich et al. (17) described similar cases of esophageal metastasis into the adrenals, and both recommended surgery as a curative treatment for this disease. However, in the case of our patient due to the degree of metastasis and the systemic symptoms, surgical resection was not recommended and our treatment goals had to shift to maintain as much of the patients quality of life as possible. The presentation and degree of metastasis of our patient as well as the in-eligibility for surgical resection provided a unique challenge that should be further investigated. “Adrenal Incidentalomas” as Doesburg et al. (18) describes have an unknown occurrence and significance rate in the setting of esophageal cancer. While many similar case reports describe resectable tumors, the unique challenge of advanced esophageal disease with the addition of such extensive metastasis proved to be an unforeseen obstacle to treatment.

Future studies should explore chemotherapeutic agents that can target advanced esophageal cancer while also successfully targeting progressive metastasis. Future studies should also focus on the patient’s overall health and quality of life. A multidisciplinary team may be instrumental in helping patients through the challenges of systemic therapies in hopes they could tolerate a more aggressive treatment approach then current literature describes.

## Conclusion

While aggressive treatments may offer hope for a cure in select patients with isolated adrenal metastasis from esophageal cancer, the general approach should remain cautious, with systemic therapy as the first line of defense. This case highlights areas where ongoing research and clinical trials are needed to better define treatment protocols and improve outcomes for this challenging patient group.

## Data Availability

The original contributions presented in the study are included in the article/Supplementary material, further inquiries can be directed to the corresponding author.
