# Supplementary material for: Case Report: Solitary adrenal metastasis from esophageal adenocarcinoma
Source: Front Med (Lausanne). 2025 Jul 17;12:1623443. doi: 10.3389/fmed.2025.1623443 (PMC12310622; doi:10.3389/fmed.2025.1623443)

Supplement Table: Investigations of the Patient

Test	Result
CEA	6
Adrenal gland biopsy	Metastatic adenocarcinoma
CEA	3.4
PET/CT	No evidence of hypermetabolic mediastinal/axillary lymphadenopathy/lung nodules, mild activity distal esophagus SUV 4, prior left para esophageal node resolved. Prior right adrenal activity resolved, SUV 1.9.

Supplemental Images: Series of PET-CTs

Image 1, Staging PET-CT Scan (Week 1)

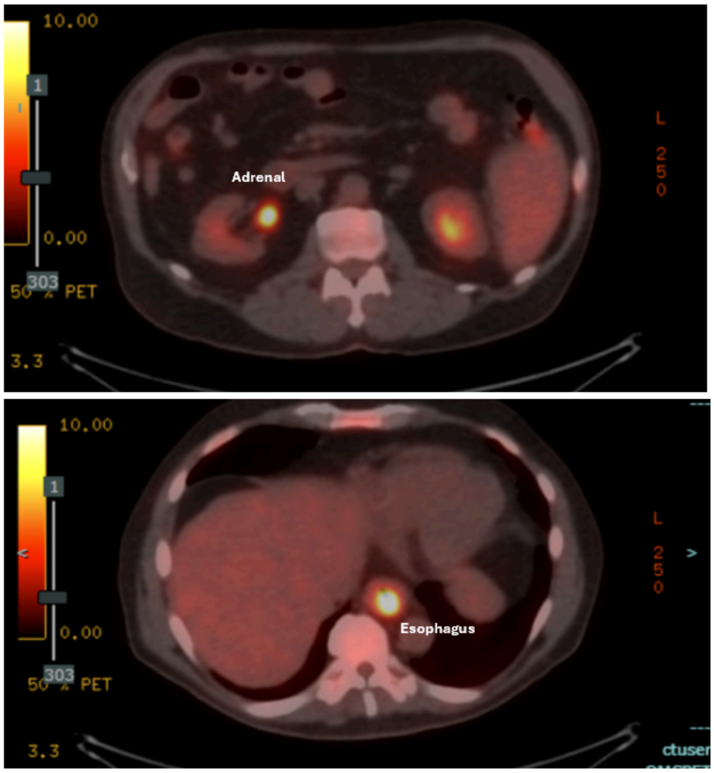

Image 2, PET-CT Scan (Week 14) after three months of FOLFOX + Dexamethasone

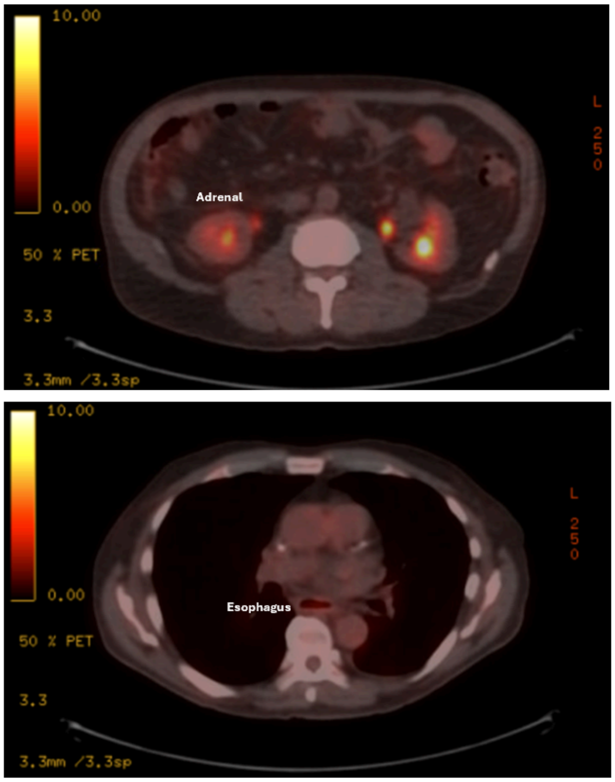

Image 3, Interval PET-CT (Week 25) with new and increased hypermetabolic activity

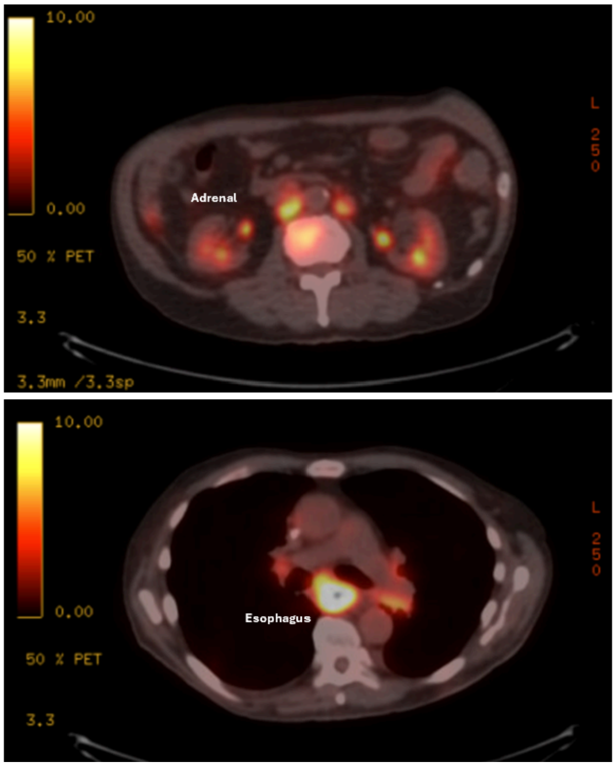

Supplement: Supplementary file 1 [file Data_Sheet_1.pdf]
